# Supplementary figures and images for: SF3B1K700E mutation in human embryonic stem cells causes aberrant expression of immune-related genes
Source: PLoS One. 2025 Dec 17;20(12):e0334361. doi: 10.1371/journal.pone.0334361 (PMC12711085; doi:10.1371/journal.pone.0334361)

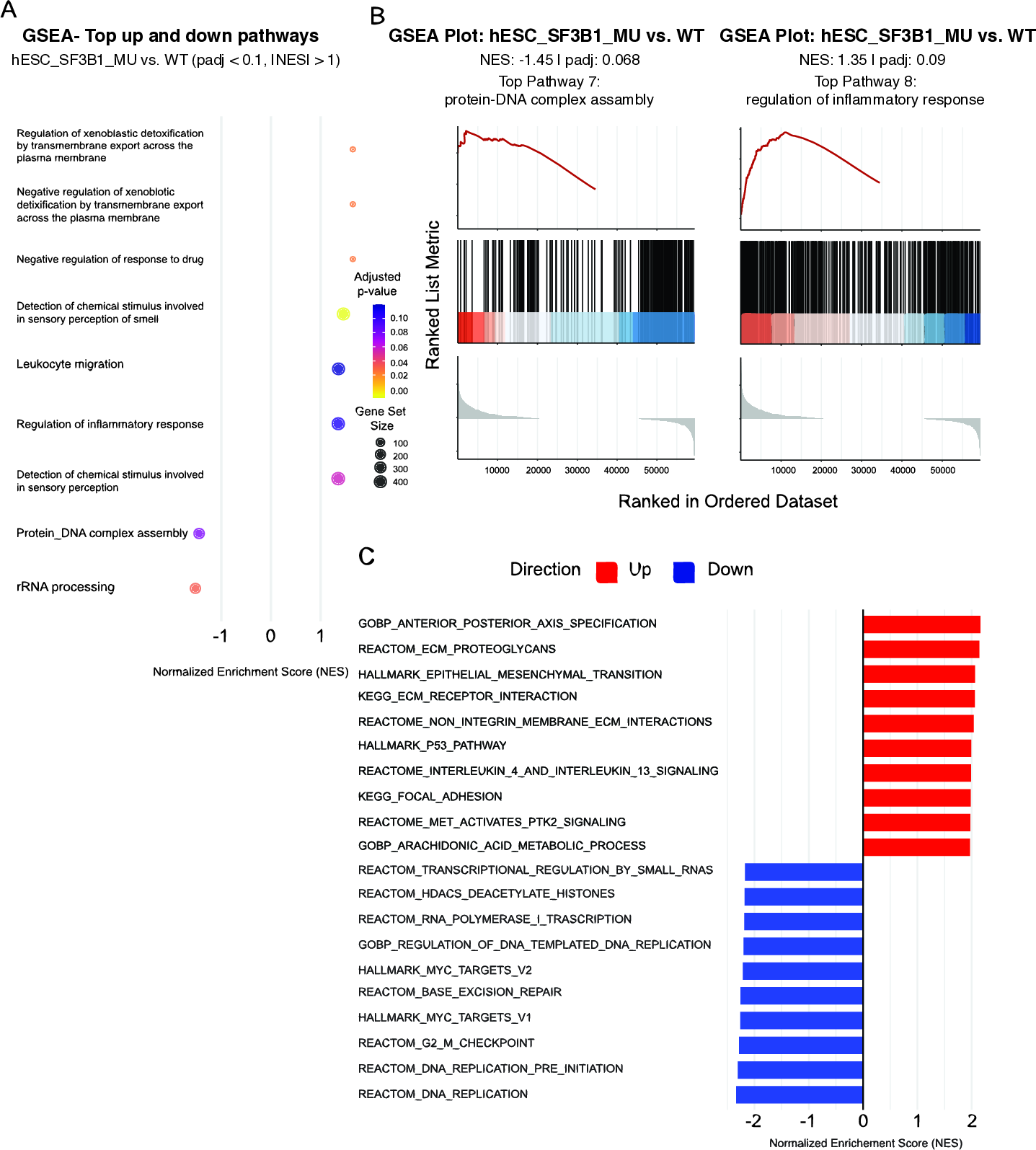

Supplement: S1 Fig — (A) Bubble plot summarizing the top enriched biological pathways among differentially expressed genes. The x-axis shows the normalized enrichment score (NES), with positive values indicating upregulation in SF3B1_mutants and negative values indicating downregulation. Bubble size reflects gene set size, and bubble color encodes the adjusted p-value (padj), only pathways with padj < 0.1 and |NES| > 1 are shown. (B) Representative enrichment plots for two leading pathways. Left, protein–DNA complex assembly (NES = −1.45; padj = 0.068) is negatively enriched in SF3B1 mutants. Right, regulation of inflammatory response (NES = 1.35; padj = 0.09) is positively enriched. For each plot, the running enrichment score (top, red) is displayed across the ranked gene list (middle barcode plot; red = up in SF3B1_mutant, blue = down), with the rank metric distribution shown below. (C) Bar plot of selected hallmark, GO, and Reactome pathways ranked by NES. Red bars show pathways upregulated in SF3B1_mutants; blue bars denote downregulated pathways. The x-axis shows NES. (TIF) [file pone.0334361.s001.tif]

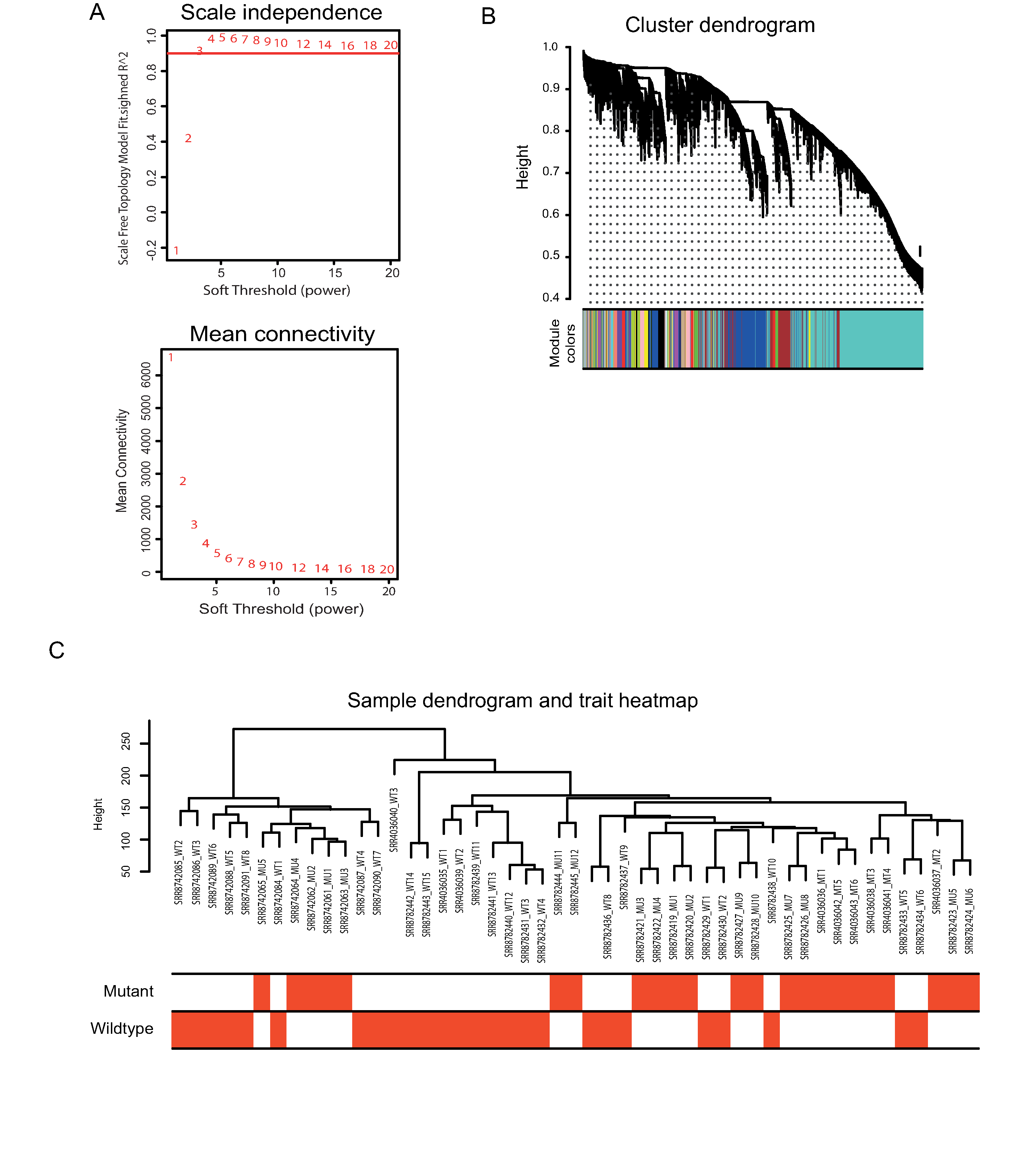

Supplement: S2 Fig — (A) Determination of soft-thresholding power in WGCNA. Scale-free topology index and mean connectivity for each power are shown. In this study, the threshold was reached for a power of 3. (B) Dendrogram of differentially expressed genes clustered based on the difference metrics (1-TOM). (C) Sample dendrogram and trait heatmap based on expression data from 3 MDS datasets. (TIF) [file pone.0334361.s002.tif]
